# Women academics and the changing psychological contract during COVID-19 lockdown

**DOI:** 10.3389/fpsyg.2022.940953

**Published:** 2022-08-11

**Authors:** Linda Ronnie, Marieta du Plessis, Cyrill Walters

**Affiliations:** ^1^School of Management Studies, University of Cape Town, Cape Town, South Africa; ^2^Department of Industrial Psychology, University of the Western Cape, Cape Town, South Africa; ^3^Department of Education Policy Studies, Stellenbosch University, Stellenbosch, South Africa

**Keywords:** psychological contract, women academics, lockdown, pandemic (COVID-19), higher education, South Africa

## Abstract

This study examines the psychological contract between academics and their institutions during a time of great stress—the COVID-19 pandemic. Given that relationships between these parties have been found to be deteriorating prior to the pandemic, we believed it pertinent to explore how environmental changes brought about through lockdown conditions may have shifted the academic-institution relationship. Through a qualitative research design, our data is from 2029 women academics across 26 institutions of higher learning in South Africa. The major shifts in the psychological contract were found to be workload and pressure, provision of resources, top-down communication, as well as trust and support. Whilst these shifts altered the transactional and interactional nature of the psychological contract, violation, rather than breach, occurred since the emotional responses of participants point to incongruence or misalignment of expectations between academics and their institutions during this time of crisis. We offer recommendations for rebuilding trust and negotiating the psychological contract to re-engage academics in the institution.

## Introduction

Higher education institutions worldwide have been affected by the COVID-19 pandemic with resultant campus closures to enforce social distancing measures (Toquero, 2020). South African institutions of higher learning were compelled to identify and implement various strategies that contributed to sustaining the academic project, which included but were not limited to engaging in emergency remote learning and teaching, working from home arrangements for staff, finding alternative ways to support students, and reallocation of budgets to address the emerging needs. The psychological contract between academics and their institutions also appears to be shifting during this time of great stress (Kowal et al., 2020), although relationships between these parties had already been found to be deteriorating prior to the pandemic (Bolden et al., 2014).

Over the last two decades, structural changes in higher education have impacted on the work life of the academic. Most significantly, there has been a shift from universities providing elite education to providing mass education, referred to as massification (Altbach et al., 2009; Adcroft and Taylor, 2013; Altbach, 2015). At the same time, managerialism has taken root within universities, affecting the way they operate (Churchman, 2002; Altbach, 2003; Smeenk et al., 2006). The rise of managerialism requires academics to work and behave like corporate employees, accountable to measurable performance management targets (Winter, 2009). Consequently, the balance between research and teaching duties has shifted, with greater demands placed on research output and fundraising (Harris, 2005; Billot, 2011). Working in this environment, academics have also seen an increase in administrative requirements from both government and management, increasing the academic workload and frustration (Tight, 2010; Parker, 2011).

More broadly, the rise of neoliberalism, marketization, and privatization in higher education has seen external stakeholders placing new demands on universities (Harris, 2005; Bundy, 2006; Winter, 2009). In particular, marketization has forced academics, who are already coping with increased workloads, to become more self-motivated, entrepreneurial, and adaptable (Morley, 2003). In the midst of this transformation, government expenditure on higher education is decreasing, even as many governments still control universities *via* research funding and accreditation requirements (Parker, 2011; Webbstock, 2016). Taken together, these changes have led to an increase in the diversity of the academic role, with consequences for what used to be a singular academic identity (Churchman, 2006). Academics are now responsible for obligations to their disciplines, their need to create knowledge, their desire to teach well, the state demand for employable graduates, and entrepreneurial activities that take advantage profitable local and global market-related opportunities. These increased demands and shifts have had the effect of reducing the autonomy of academics (Parker, 2011; Altbach, 2015). Managerialism and reduced autonomy have impacted negatively on the trust, morale, and commitment of academics with respect to their institutions (Ladwig et al., 2014; Shrand and Ronnie, 2019).

The shifts in the higher education sector over the past few decades have thus impacted the role, identity, and morale of the academic, both globally and in the South African context. Pertinent to our study are the claims that academic institutions are gendered environments (Acker, 1992) and that universities governed by managerialist agendas strengthen rather than reduce inequalities along gendered lines (Parsons and Priola, 2010). These gendered differences have been found in recruitment and selection processes and promotion practices (Van den Brink and Benschop, 2012); in the increasing demand of academic service to students (Davies and Thomas, 2002); exhausting administrative and pastoral duties (Acker and Armenti, 2004); and in the devaluing or underestimation of the academic endeavors of women (Knobloch-Westerwick et al., 2013). Sims (2021) argues that the managerialist agenda—a masculinist-dominated one, in her opinion—has shaped the choices of women academics, compelling them to either accept it, and therefore their lesser status, or embark on changing the *status quo*.

Importantly, as Bailyn (2003) commented, the “ideal employee” continues to be seen as having few interests or commitments outside of the academy. In this new environment, academics have been obliged to spend extended periods at work, including taking substantial amounts of work home (Walters et al., 2021). As a result, the domestic environments have been disrupted, causing women academics, who are often more involved in the care of dependents, to suffer more adversely than their male colleagues (Elg and Jonnergård, 2003; Barry et al., 2006). These views give rise to the focus on the academic-institution interface and what this might mean for women academics who are frequently aware of gender challenges and must either find a work-around or be side-lined by the prevailing institutional culture (Teelken and Deem, 2013).

It is therefore clear that the psychological contract between academics and their institutions has been changing over the last decade (Shen, 2010). When coupled with the COVID-19 pandemic in South Africa, there may be long-term effects on retention, productivity, and overall performance (du Plessis et al., 2022). Lopez and Fuiks (2021) have challenged researchers to investigate whether and how psychological contracts have shifted or been breached; which elements need updating, e.g., work tasks, schedules, and performance appraisals; how academic psychological contracts can be clarified and negotiated during this time of uncertainty and crisis; and how anxiety and burnout can be reduced while optimism and organizational commitment are sustained. Our paper responds to this challenge by examining the psychological contract from the perspectives of women academics in South Africa at a turbulent time for universities. This study explores a timeous issue that is currently pressing in higher education, and likely to increase in importance as pandemic-era changes carry into the future.

Through a qualitative analysis of the critical experiences of women academics during the lockdown period of the COVID-19 pandemic, as reported in an online survey, this study aims to detect changes that may be currently occurring to the psychological contract in order to understand how higher education institutions can respond and mend the contract, if appropriate. Whilst the unexpected circumstances of the pandemic-related lockdown provide contextualization to our findings and explain the causes of the shifts in practice, the experience of the psychological contract between academic women and the employer was affected.

## Literature review

### The psychological contract

The psychological contract—first conceptualized by Argyris (1960) as the mutual expectations between employee and employer—is best described by Rousseau (1989, p. 123) as “an individual’s beliefs regarding the terms and conditions of a reciprocal exchange agreement between the focal person and another party.” The concept therefore exemplifies the relationship between employee and employer and encompasses the beliefs that each party holds of the relationship (Hui et al., 2004). However, because each party holds their own perceptions, the contract may be understood uniquely by employees and employers despite the belief that obligations and commitments exist between them (Rousseau, 1995). Significantly then, individual choice—through increased or decreased engagement—underpins the psychological contract. Exchanges that transpire over a considerable period, combined with an understanding that the employment relationship will continue almost indefinitely, give rise to beliefs premised on explicit promises and elements that both the employee and employer take for granted (MacNeil, 1985). These beliefs may increase the dependence of both parties on the relationship (Rousseau, 1995). A central key issue, however, is that the psychological contract is reliant on a belief that promises, whether implicit or explicit, have been made and that a consideration has been made in exchange for it (Rousseau, 1989). This belief connects the employee and employer to a set of reciprocal obligations and responsibilities.

### Types of psychological contracts

The psychological contract has been categorized into three main types: transactional, relational, and balanced (Robinson et al., 1994; Rousseau, 1995). The fourth type—transitional—is not a psychological contract type *per se* but rather a cognitive state that mirrors an absence or breakdown of the exchange relationship between the two parties without specific performance terms (Hui et al., 2004). Table 1 highlights the features of the three key types based on two crucial dimensions—timeframe and performance terms—of the employee-organization relationship.

**TABLE 1:** Types of psychological contracts.

	Specified performance terms		
**Short term duration**	***Transactional contract***		
	Low ambiguity		
	Easy exit/high turnover		
	Low member communication		
	Freedom to enter new contracts		
	Little learning		
	Weak integration/identification		
	
	**Dimension**	**Employee commitment**	**Employer commitment**
	Narrow	Perform a fixed, limited set of duties only. Only does what they are paid to do	Offers limited involvement in the firm and little training or development
	Short term	Employee has no obligation to remain with the firm. Committed to work a limited period of time only	Employer has no obligation to future commitments. Offers a limited period of employment only

**Long term duration**	***Relational contract***		
	High member commitment		
	High effective communication		
	High integration/identification		
	Stability		
	
	**Dimension**	**Employee commitment**	**Employer commitment**
	Stability	Remain with the firm and to do what is required to keep job	Offer stable wages and long term employment
	Loyalty	Support the firm, manifest loyalty and commitment to the firm’s needs and interests. Be a good organizational citizen	Support the wellbeing and interests of employees and their families

**Long term duration**	***Balanced contract***		
	High member commitment		
	High integration/identification		
	Ongoing development		
	Mutual support		
	Dynamic		
	
	**Dimension**	**Employee commitment**	**Employer commitment**
	External Employability	Develop marketable skills	Enhance employee’s long term employability inside and outside firm
	Internal advancement	Develop skills valued by current employer	Create career development opportunities within the firm
	Dynamic performance	Perform new and more demanding goals that may change to help the firm remain competitive	Promote continuous learning and support employee in executing performance demands

Adapted from Rousseau (1995).

Recent studies have suggested an evolution in psychological contracts in terms of contract makers, location, and timing (Ashford et al., 2007; Alcover et al., 2017; Baruch and Rousseau, 2019; Griep et al., 2019; Knapp et al., 2020). While the original conceptualization by Rousseau (1989) of an exchange and reciprocal relationship still holds true, research indicates that employees and employers may have differing views of its terms and the degree to which the other party has fulfilled their obligations (Coyle-Shapiro et al., 2019).

### Breach and violation

Many, if not most, employees experience a breach of their psychological contract in the course of their employment (Montes and Zweig, 2009). Morrison and Robinson (1997) explain that these perceptions of breach come about through the misalignment between what is understood to have been promised and what is actually delivered. In essence, it is a failure of the organization to fulfill one or more of their obligations. Breach is, according to Conway and Briner (2005, p. 64), “subjective and based on a perceived rather than an actual agreement, and can occur in relation to any explicit or implicit promise.”

Pate (2006) describes three triggers of psychological contract breach—distributive, procedural, and interactional aspects of justice—as a result of an organization’s inability to meet their obligations. “Distributive breach occurs when outcomes are perceived to be unfairly distributed for example, financial rewards. Procedural breach refers to the perception of the unfair application of procedures, such as promotion. Finally, interactional breach is linked to employees’ perception of trust of superiors and the organization as a whole and occurs if employees feel they have been treated badly” (Pate, 2006, p. 34).

Literature points to a distinction between violation and breach where violation captures the emotional response that may arise from breach itself. As Pate (2006, p. 36) explains, “[b]reach is therefore confined to a calculative identification of injustice but at this stage emotional responses are not engendered.” Incongruence and reneging are two types of violations of the psychological contract (Conway and Briner, 2005; DelCampo, 2007). Incongruence occurs when there is a difference in perception or understanding between the employee or the employer. Reneging transpires when either party knowingly breaks a promise which may occur purposively or come about through unforeseen circumstances.

### Academic psychological contracts

Universities, through their leadership, need to understand their role in shaping the academic-institution relationship. If the psychological contract which underpins this relationship is well managed, this is likely to have positive implications for all parties as it has been shown to positively influence motivation, performance, and commitment (Rousseau, 2004; Walker, 2013; Conway et al., 2014). In contrast, breach of trust or failure to deliver on expectations have been shown to have a lasting negative impact on such attitudes and behaviors (Jensen et al., 2010; Griep and Vantilborgh, 2018).

A cascading effect can also take place when psychological contracts are fulfilled or breached (Bordia et al., 2008). In the context of higher education, if middle managers—as in Deans and Heads of Divisions/Departments—believe that the university has reneged on promises regarding health and safety and online learning support, this could have a negative trickle-down effect on academics, potentially influencing their interaction with students and consequently their learning (Lopez and Fuiks, 2021). Alternatively, if promises are fulfilled, a positive cascading impact may occur, likely resulting in productive outcomes, such as promotion and career advancement for researchers and concomitant benefits such as increased research productivity for universities (Dabos and Rousseau, 2004).

There is also the idiosyncratic and active nature of the psychological contract. As the contract is based on perceptions between the parties, each psychological contract is specific and distinctive to each individual (George, 2009). Studies have shown that academics desire recognition and treatment as professionals and have expectations of being recognized for their commitment to the academic profession, the university, and their students (O’Neill and Adya, 2007). Mousa (2020) noted the role of responsible leadership in mediating the relationship between the perceptions of inclusion felt by academics and their psychological contract type. According to Shen (2010), transactional contracts were more prevalent amongst academics and their expectations of university management were focused on the provision of a safe workplace, resources to conduct academic work, equal and competitive pay, and a reasonable workload.

Results also showed that differences across gender “did not demonstrate significant influences on the perception of the psychological contract fulfillment, except that male staff tended to be more satisfied with the fulfillment of reasonable workload” (Shen, 2010, p. 586). However, a possible explanation offered for this was that women may have felt the current workload unfair given the likelihood of more family responsibilities than their male counterparts. Differences across gender in terms of psychological contract fulfillment have been found in a variety of settings such as the public sector (Morley, 2003); manufacturing (Abela and Debono, 2019); healthcare, public administration and business (Kraak et al., 2018); and the service sector (Karani et al., 2022). However, there is a paucity of research that focusses on the psychological contracts of women in the academic profession. Our study addresses this gap in the literature.

## Methodology

Using a purposive sampling approach, we selected women academics at higher education institutions countrywide to participate in a larger survey study about the experiences of women academics in South Africa during the COVID-19 lockdown period, which lasted from March to September 2020. During this period, universities were closed for all in-person classes and activities, and all staff and students were required to work from home. We sought the experiences of women in particular as research has shown that, in South Africa, the role of childcare, homecare and care for parents typically falls to women (Hatch and Posel, 2018; Casale and Shepherd, 2020). We therefore argue that women provide a far richer and deeper understanding of changes to the psychological contract.

### Sampling

The study aimed to survey females in academic roles at South African universities: researchers, lecturers, professors, faculty, and adjunct faculty. Working with the universities to either access their mailing lists or share the survey link, a survey was distributed to the entire population of female academics at each institution. Our sample consisted of 2029 participants from 26 institutions. Information about participants’ positions, discipline, departments, or research areas was not collected to ensure confidentiality.

### Data collection

The survey sent to female academics consisted mainly of Likert scale questions and one open-ended question. This study focuses on the open-ended responses the survey. Drawing on the critical incident technique (Flanagan, 1954, cited in Bryman and Bell, 2018), participants were asked to share stories of their experiences and specific situations that were significant (“critical”) to them while working during the lockdown period. This line of enquiry is in keeping with Herriot et al. (1997), who argued that few employees would provide pertinent information about their expectations of their employer directly. Upon review of the 2029 responses, 494 participants highlighted and discussed the relationship with their manager and/or institution. This narrowed the data set for qualitative analysis.

### Data analysis

Thematic analysis was used to analyze the critical incidents. An inductive thematic analysis was conducted using the procedures by Braun and Clarke (2006), which include familiarizing oneself with the data, generating initial codes, searching for themes in the incidents, reviewing the themes, and defining the themes. The first two authors conducted the thematic analysis, while the third author acted in a data validation role to review and check the interpretations of the other authors.

The dependability of the study was ensured during the code generation, theme identification, and review processes, first by extracting the exact phrases and words of participants in their descriptions of “critical” experiences for use in the findings and analysis (Given, 2008). The analysts reviewed these incidents for common themes or recurring phrases, sentiments, ideas, and concepts. The validity and reliability of the findings, in terms of the meaning of these excerpts, were confirmed using a code-recode procedure as well as peer examination between the data analysts to ensure the consistency of the findings. Once the findings were consistent with the data throughout, they were documented and reviewed by the third author as an addition validation measure. During this stage, alternative interpretations were raised and clarifications to the themes added where needed (Leedy and Ormrod, 2019).

### Ethical considerations

Ethical clearance for the project was granted by the University of Stellenbosch. Individual access was negotiated with each university. To protect the privacy of individuals and their institutions, no identifying demographic information has been used in the reporting of the findings.

## Findings

The experiences and stories shared by participants about their managers and institutions constitute different types of “shifts” in their overall working experience compared to pre-pandemic conditions. We discuss the various shifts and their impact on the psychological contract of participants: workload and pressure, provision of resources, top-down communication, and trust and support.

### Shift 1: Increase in workload and pressure

Participants experienced a shift in the way work was done, i.e., the need to provide emergency remote teaching and working from home, but more so the volume and intensity of work changed. Participants noted:

The basic academic expectations have not changed to cope with the pandemic, but rather have increased to include new items which reduces research time significantly. [P1445]

During the weeks of lockdown […] I was extremely tired, more so than what I was working on campus. Between administration work and lecturing and getting ready for online mode was exhausting. Therefore during lockdown […] I hardly was able to even sit behind the microscope. [P1123]

Teaching online resulted in more time needed for preparation of teaching and learning material. [P533]

Participants needed to adapt to the shift in work, with the clear consequence being that hours required to complete the work increased. For instance:

During lockdown and up until now I am often working 10–12 hours daily just to deal with teaching, admin, responding to student, student referral (emotional labour with many distressed students) and last minute instructions to change/amend plans. [P1251]

My daily workload has increased from before 8 to 9 hours to now between 12 and 13 hours. [P1312].

Not only were more hours required, but participants experienced the expectation of being available outside of traditional working hours.

My day never ends as students’ WhatsApp questions till late in the night—last night till 1am! There are no boundaries. Meetings go on till 7 p.m. at night sometimes even later. [P42]

Students also want help over weekends, after hours and even at night, so there is never a break at all. [P216]

This notion of being available at all hours of the day was reinforced not only by university management, but through understanding the plight of students who received limited data for educational purposes. Participant P1976 explained:

The students are given 30 gigs a month. 10 G are anytime, the rest are night owl (after midnight and before 6 a.m.). This has made data-dependent online teaching (live teaching, Zoom with video) all but impossible. The only colleague I know of who has “lectured” after midnight is a single woman with no children. [P1976]

Whilst the COVID-19 pandemic presented unusual circumstances, organizational expectations of performance were upheld.

I have two children. All of a sudden both of them had to be home schooled. I felt that the [the university] gave no support to this situation that many parents (not only mothers) found themselves in. Our performance management continued as usual, with no seeming understanding for this situation. I told my line manager that my days have been cut in half, but other than a sympathetic acknowledgement, it changed NOTHING with regards to what was expected of me. [P1553]

… the institutional message in this regard, also included an explicit reference to the institutional disciplinary code and procedures. The implied message is that if you don’t sustain a high level of academic productivity despite the lockdown, you will be disciplined. Such messages makes the institutional commitment to staff wellbeing (and the money spent on it) null and void, and it flies in the face of an ethic of care. [P106]

I feel bombarded with too many invitations to training for Teams/Moodle/other support, and repetitive requests for reporting on various aspects of work. It’s as if management imagine that we are not gainfully occupied during this time and feel the need to constantly chivvy us. [P1267]

The responses from participants point to the organizational expectation of performance was not lowered, whilst even more tasks and work hours were required. Whilst most participants seemed to do what they can to adapt to the increase in workload, the consequences of such efforts came to light. For example:

Through protests and pandemics we are just told that we need to complete the year without any consideration of our wellbeing. [P1553]

I don’t know how we are going to manage completing this year without suffering with burnout or having a mental breakdown. There is no physical, emotional or psychological support for what we are having to endure. [P1803]

Participants experienced the breach of increased workload with no additional support and recognition of circumstances as a violation of the psychological contract. This is evident in further responses from participants:

It feels like management is always asking more and more from us and offering nothing in return. [P2116]

To be honest I believe the support from the university was sorely lacking and no care or concern was shown for the academic staff who were teaching in this block, with almost 24/7 working. [P418]

I often felt like resigning and I did feel unsupported. [P305]

There is very little sympathy from senior management for the workload placed on working mothers during any type of crisis—and the pandemic brought that into clear focus. Teaching and meetings are difficult when you have to share resources e.g., computers, wifi, space etc. [P313].

### Shift 2: Provision of resources

Shifting from an office-bound work environment to working from home presents many challenges for organizations. Amongst these challenges are the provision of physical resources (i.e., computers) and timeous capacity development. Our participants experienced that their institutions were slow to respond to the need for increased resources. For example:

I feel very let down by my university. I ended my face-to-face lectures on the Friday and was expected to miraculously move everything online with no data, no laptop and no support the following week. [P1942]

Some colleagues had trouble with accessibility and technical issues that hindered their productivity, I am fortunate to have all the resources at home that I pay for myself (fast internet, etc.). [P763]

The lockdown showed the kind of management we have. I believe management could have done better to ensure that productivity is high, for example I had to use my own laptop as the institution I am at did not provide any laptop to me. If I did not buy my own laptop I was not going to be able to work. Secondly, I also had to get a work desk and table to work on, I had to buy my own data. [P1617]

Many participants, like P1942, P763, and P1617 above, defaulted to using their personal equipment and resources to continue with their work. However, dissatisfaction started to emerge as this became the norm.

I feel that I was not provided with sufficient resources in terms of laptop, printing equipment, etc., and I would need to fork out of my own pocket without the possibility of reimbursement. [P1344]

Having to depend on a university that did not have technological gadgets in place and having to improvise using my personal resources and skills was emotionally draining. [P1664]

The need for resources was, however, not limited to physical resources such as laptop computers, printing equipment, and microphones. Participants expressed how the inadequacy of administrative and technical support further exacerbated time constraints. For example:

The administrative load was relentless as we had no help at all from administrators who would usually assist with some student queries, assignment submissions, mark recording and managing class groups. [P1251]

Most of the time was spend on student queries and it would to some extent be difficult to promptly respond as resources were not adequate. [P690]

The amount of pressure from [my university] during COVID-19 was overwhelming. More was expected with less resources. [P948]

Participants also mentioned the lack of personal and organizational resources to assist students to deal with emotional burdens resulting from the students’ own challenges. In many cases, students lacked adequate resources for learning (specifically data and devices). Participant P1714 explained that whilst the lecturer is not responsible for assisting students with physical resources, students still expected answers and accommodation in terms of deadlines from lecturers.

The students vented all their frustrations on us and the lecturers were just told to deal with it as best we can, not a fair situation as most academics are not trained in providing emotional support in any way. [P1714]

Time boundaries have been erased as my students contact me any day, any time and I feel compelled to respond immediately because they are probably struggling more than I am. [P1147]

The biggest challenges that I experienced are (1) the students needed much more support, we went into multiple modes of connecting with students, even using my personal social media accounts, encountering students who would need support 24/7, providing data at my own cost to students, and converting everything to synchronist teaching); (2) reaching out to the “missing” students; (3) I have to use my own internet and data (the university did not provide this to academic staff); (4) working off campus now places higher demands on me being available at longer hours even over weekends. [P663]

Therefore, whilst the organizational expectation was for staff to work from home and adapt to emergency remote learning, participant’ expectations of receiving adequate physical and human resources to do so was not met. This required participants to be innovative to find solutions. However, this was emotionally taxing and was experienced as a violation by participants as they expressed unfairness in needing to deal with the situation without adequate support from their universities.

### Shift 3: Confusing communication from the top

Whilst participants commented broadly on the communication from university executive leadership, communication from line managers was an important factor. For instance, Participants P71 and P166 commented:

I do have a very supportive line manager and he has encouraged me throughout the lockdown. [P71]

I am also grateful for an understanding and supportive manager who constantly checked on us and guided us on working effectively during these unprecedented times. [P166]

However, during times of crises, employees require direction and clarity from the leadership of the institution. This was, however, not the experience of study participants from all the institutions.

The leadership did not provide timely direction. [P1164]

Mixed, ambiguous confusing messages, change of assessment methods without training. In summary: HELL. [P1839]

The messages from top management changed and we had to redo a lot of work. [P673]

I would have liked and expected to see more communication coming from all managerial levels above me (from HoD, to mostly Dean and upper management), as well as from HR in terms of checking on us employees about our wellbeing. [P481]

Even for those in leadership positions at the faculty level, there was also a sense of frustration:

This lockdown was incredibly time-consuming as we were at the frontline of dealing with student and staff queries [and I found] the incredible increase in admin as [a head of department] overwhelming and exhausting. [P67]

The constantly changing environment and the emotional toll of being a head of department has been overwhelming. It means I need to support and help staff in the department, whereas support from my managers are missing as they are probably running around attending to “more important matters” than their middle management. [P111]

I often feel overwhelmed at the sheer scale of the academic work—having to finish the academic year and support others to do so in my management role. […] Giving input on normal university or sectoral matters like policies seem a distraction from completing the year and I wish that this could be the sole focus. [P127]

I am in management so the lockdown has been a nightmare. We work day and night and during weekends, I feel drained at the end of the day. Over and above the normal meetings and reports, the Lockdown meant shutting the university completely and thereafter planning to open under restricted conditions—we had almost daily meetings for the COVID issues, moving budgets, and ridiculous amounts of reporting. [P195]

As universities shifted to online learning and staff members working from home, communication shifted exclusively to digital means, as participant P1089 explained.

The loss of interaction with fellow colleagues, academic facilities/resources and support is one big negative aspect of the lockdown. The additional burden of home schooling has also been a big derailer. And even though technology bridges the gap to a large extent, the inability to have one-on-ones with supervisors, team/group members, friends and family has been brutal. [P1089]

There were also elements of increased managerialist behavior in terms of reporting, additional meetings, and top-down decision making.

Management seem to be disconnected from the problems experienced on the ground and their decisions create more work, stress and uncertainty instead of reducing it. [P153]

The main difficulty I experienced was having to get onboard very quickly with online teaching, and also dealing with an overwhelming amount of instructions, materials, courses generated by management. [P277]

There could be so many ways in which the university could have lightened its load on academics, but endless demand for reporting, surveys, routine ‘business as normal’ things like School evaluation reports continued as normal. Monthly meetings now needed weekly—and I spend hours every day in admin and management meetings that are really reporting meetings. [P285]

Academics were told to do things we knew would not work, but we were not only ignored, but instructed (as if we were robots) to implement whatever management dreamt up overnight. Naturally this was a complete disaster and management even had to go onto [radio] to apologise. [P658]

I was bombarded with emails from management that showed no respect for our time. Countless reports had to be written and I felt it was a way of management controlling. [P664]

The impact of lockdown was significantly more challenging in the absence [of] strong, communicative and forthright management structures. Email is not sufficient communication; it suggests authoritarian undertones and telegraphs apparent tone-deafness. Top heavy structures are failing students on mass. [P1515]

Whilst most participants adapted to this shift, a breach was experienced as the content and frequency of the communication were not always aligned to needs.

Management still wants everything to be done, but don’t talk directly to those staff members who are not doing their work. It’s very frustrating and unfair. [P1916]

The university management did not send the information that was sent out to the students to the lecturers also —as if the lecturers do not need to know that. However, when the students have problems they actually turn to us as lecturers, and if we are not informed then it takes us extra time to find out how they can be helped. [P30]

The communication received from the institution also negatively impacted my emotional wellbeing. Example, one email we received regarding our leave reflected the fact that academics had leave during the initiation of lockdown. Yet, my colleagues and I were working trying to save the term from the resultant student protests. I am in the second week of ‘leave’ for the break between semesters, yet I am spending my time preparing for a subject I have never lectured before. We are at the risk of serious burn out. [P1553]

I am tired of asking for support from my faculty, [head of department] and line manager, as well as our online learning team as I am ignored. [P1942]

I have been in a single (group) meeting since the lockdown in March, and have had no individual meetings with my line manager at all, only a single 5-minute phone call…it leaves me kind of directionless, as a young academic. [P253]

These responses from participants indicate a misalignment between what was promised and what was delivered. In some cases, participant experiences indicate experiences of violation as the organization reneged on what had been considered fair practice.

### Shift 4: Trust and support

As much as academic staff were ill-prepared to engage in emergency remote teaching, most managers were ill-prepared to manage staff in a virtual manner. Participants expressed different opinions on how they experienced trust from their managers.

The lack of trust from Deans/Academic Heads was disheartening. Lecturers were not ‘taking a holiday.’ Many were working excessively more during this period to complete academic work and the sudden excess of admin reporting. These various aspects did not even start to factor in family care. [P1755]

It was great to know that my line manager trusted me. He never breathed down my neck trying to see what I was doing. [P1332]

The theme of trust and support was also most closely aligned with gendered expectations.

The lockdown has emphasised for me how out of kilter my faculty and institutional leadership and managers are with the realities of their broader workforce—particularly that of women. The running narrative within my faculty has been centrerd around that working from home is a luxury and a privilege (as you are supposed to have more time not having to travel to and from work), and (as a result) you are supposed to be academically more productive. Those in powerful positions do not have small children. [P106]

Some of my colleagues and managers don’t have a family or children and they boasted about how much work they got done at home and how productive they were during the lockdown. I am usually very efficient and productive at the office, but my productivity decreased drastically during the lockdown. I was so exhausted from domestic responsibilities and balancing meetings and other ‘admin’ that I couldn’t keep it up. [P1451]

It brought tension in the household and in time lead to deterioration of my relation with my husband (soon to be ex-husband) …The personal circumstances brought by the lockdown associate with a very bad management in the work place lead to demotivation and taking strain every day. [P619]

The responses indicated that academic women and parents may have needed additional support.

The lockdown has caused a lot of stress for the academic staff in my department, especially those with young children. They have required a lot more emotional support from me, as their line manager, than under normal circumstances. [P326]

In addition to being an academic and in a leadership position within my department, I am now also a housekeeper, a child carer, a primary school teacher (for which we as parents are both ill-equipped), and a support structure for the extended family affected by the pandemic—including aged and vulnerable parents. Even though I have a very supportive partner, I am still the primary carer. There has been no institutional support or much recognition for this. [P106]

At the start there was confusion of how to proceed and lots of uncertainty. Lack of support from management and poor communication. Management increased anxiety with rumours concerning job loss, leave without pay and work arrangements. There was more concern about financial impact on the department than about safety of staff. [P154]

It is clear that participants in our study expected more from organizational leaders at all levels. This points to interactional breach, leading to a violation of the psychological contract. Not feeling trusted, inadequate communication, and lack of recognition left participants devalued, disheartened, and less committed to their institutions. For example:

I see this lack of communication and clarity in guidance as a fail in leadership, and I actually carry some resentment towards the management of my University for not performing this very important task of leadership (i.e., reaching out to employees, keeping an open line of communication, leaving us confused) during this crisis. I have definitively much less faith in our management than I had before the lockdown. [P481]

## Discussion

The psychological contract for participants in our study not only shifted during the lockdown period of the pandemic but brought to the surface the disconnect between academics and their institutions. There is general agreement that context impacts the employment relationship and therefore contextual factors also directly or indirectly influence triggers of breach (Guest, 2004; Pate, 2006). The circumstances of the pandemic—its unexpected nature and the fact that higher education institutions were simply ill-prepared for dealing with the lockdown conditions—therefore contributed to the rise in the lack of fulfillment of the psychological contract of the women academics in our study. Shen (2010) has already noted the transactional nature of academic psychological contracts and the findings of this study have highlighted the fault lines in South African universities. In particular, Shen’s study showed that having a fair and reasonable workload was one of the least fulfilled items for academics in her study. In conjunction with the sharp increase in workload brought on by the pandemic, it is not surprising that our participants highlighted this as a crucial debilitating factor.

Being denied resources and then having to provide these by using personal devices and purchasing mobile data in order to work from home appears a clear violation of the psychological contract and can be considered a failure to provide acceptable working conditions (Vantilborgh et al., 2016). This is a clear example of a shift of conditions and job expectations that are likely to contribute to increased job dissatisfaction in academia (Pienaar and Bester, 2006). Despite these challenges, women academics in our study focused on maintaining their psychological contracts with students through the provision of support, an element highly prized by students (Naylor et al., 2021).

Our participants provided numerous examples of interactional breach (Pate, 2006). In particular, they spoke to a form of interactional breach where one lacks access to information (Rousseau, 1995), in this case due to poor communication or non-transparency from university management. The lack of proper communication resulted in feeling a sense of betrayal—which has a stronger possibility of inducing higher levels of psychological contract breach (Rigotti, 2009)—and ultimately shapes poor relationships between academics and their institutional managers through perceptions of low interactional justice (Rousseau, 1995).

In many of the cases, violation, rather than breach, appears to have occurred since the emotional responses expressed by our participants point to incongruence or misalignment between the parties’ expectations during this time of crisis. Violation has been described as the “intense reaction of outrage, shock, resentment, and anger” (Rousseau, 1989, p. 129). A prime example of violation is the consequence of the unanticipated extension of the workday resulting from the shift to online teaching and the almost relentless nature of ongoing student engagement. The lack of appreciation and care, low levels of trust and support, and the negative impact on academic wellbeing were keenly felt by our participants. Past studies have found that psychological contract breach is negatively related to mental health with manifestations of increased anxiety, tension, and distress (Parzefall and Hakanen, 2010; Garcia et al., 2017; Reimann and Guzy, 2017). The middle management layer of university structures was not exempt from these experiences, with heads of departments expressing frustration, exhaustion, and feelings of being overwhelmed. The middle management experience is particularly alarming given the potential of a trickledown effect to staff (Lopez and Fuiks, 2021).

Examples of managerialism showed in the focus on organizational policies, daily reporting, increased meetings, a focus on the financial bottom line, and the like. Thus, it appears that beyond the low influence and lack of shared governance already present (Parker, 2011), a further erosion in academic autonomy took place during the lockdown phases of the COVID-19 pandemic (as confirmed in a study by Hardman et al., 2022). In contrast to universities worldwide, South Africa experiences a shortage of academic staff and, in light of that reality, it is concerning that management did not involve academics more directly but instead mainly issued top-down directives. Trust, morale, and commitment—elements all central to the maintenance of the “employment deal” (Guest, 2004)—were shown to have been negatively affected.

The effects of a lack of fulfillment of the psychological contract are not to be underestimated. Breach and violation can be considered disruptive signals and are likely to prompt negative reactions (Coyle-Shapiro et al., 2019). These include disconnection and disengagement (Soares and Mosquera, 2019), lower trust (Zhao et al., 2007), reduced job satisfaction (Raja et al., 2004), and an increase in organizational cynicism and counter-productive work behaviors (Johnson and O’Leary-Kelly, 2003; Doden et al., 2018). Uncertainty—such as the situation arising during the COVID-19 pandemic—results in more transactional and transitional contract types with an emphasis on an almost absolute economic exchange to meet an organization’s immediate talent requirements (Jeske and Axtell, 2018; Holland and Scullion, 2021). Our findings show a strong inclination toward a transitional psychological contract type, which is an employment relationship that is eroding between the parties—in this case, women academics and their institutions—creating a cognitive state due to organizational change and transition (Rousseau, 2000). This is the least stable of all contract types and is characterized by highly ambiguous expectations as the organization operates in a reactive rather than proactive manner (Rousseau, 1995). This contract type arises when the employee has reservations about the motives and objectives of the employer and is maintained when employees continue to receive confusing organizational signals (Avey et al., 2009). Fortunately, this type of contract is not typically maintained over the longer term but is likely to move to a transactional type as lockdown measures are removed and face-to-face interactions resume at universities.

### Recommendations

In light of the findings, our paper makes several recommendations, not only for women academics, but for academics more generally. The changes in psychological contract described in this study may not solely apply to academics who are female, but to any academic faced with the same set of pressures in their working environment, through some combination of environmental change and work-life demands. As the COVID-19 pandemic has reshaped the crucial relationships between academics and their institutions, we suggest what higher education institutions can do to regain and restore trust, reinforce healthier psychological contracts, begin the process of rebuilding support within the academic base, and ensure retention of their most valuable knowledge workers.

We acknowledge that the academic landscape is shifting in the post-pandemic environment and therefore changes to the psychological contract between academics and their institutions will need renegotiation. Dhanpat (2021) suggests that employers become aware of the impact a digitized workspace will have on the employment relationship. Hybrid forms of teaching are on the rise and academics will have to adapt to those realities. However, in terms of support, university management must ensure that staff are suitably supported in a manner that enables them to optimize their overall academic roles and responsibilities (Shrand and Ronnie, 2019). In addition, relationships within the workplace need to be maintained and individuals, teams, and their management need open communication, and a sense of continuity and community. When a sense of value in the relationship permeates the parties to the employment relationship, individuals are likely to experience not only a sense of self-worth but have a belief in processes and practices being fair. This is because a sense of trust has been created (Colquitt and Rodell, 2011).

In their meta-review, Kähkönen et al. (2021) describe trust as a triple-layered construct that combines psychological process and group dynamics with organizational actions at the macro-level. These authors suggest trust repair between employees and their leader–in our case, the academic and their head of department—should be considered in the broader context, i.e., as a group or a team, while trust repair at the collective level should be approached in an organizational context, i.e., between academics and the institution’s senior leadership. At a macro level, this requires more than just verbal exchanges but a more substantive, active response and co-created organizational reform to repair employee trust. Lawton-Misra and Pretorius (2021) argue that self-awareness, compassion, empathy, vulnerability, and agility are essential leadership characteristics to navigate through the crisis.

Sverdrup and Stensaker (2018) propose a trust restoration process in a three-stage model: (a) restoring reciprocity through re-establishing balance in the relationship; (b) re-negotiating the transactional terms of the psychological contract *via* clarifying the rules and expectations for future collaboration; and (c) extending and deepening the re-negotiated psychological contract to include relational terms. The model is particularly useful as it is applicable to change contexts typified by high levels of uncertainty—such as those caused by the COVID-19 pandemic. In applying this model to the academic setting, we suggest that restoring reciprocity might come about through acknowledgment that trust between academics and their institutions had broken down due to a violation of the expectations academics held of their institution. Whilst the pandemic crisis required relinquishing control in favor of more collective leadership approaches (D’Auria et al., 2020), managerialist reactions were experienced by academics such as those in our sample. Thus, university management should admit their shortcomings and apologize for those. This is a crucial first step in restoring and rebuilding trust between the parties—essentially a recalibration of the relationship—before moving on to discussion around future-orientation.

It is becoming clear that the COVID-19 pandemic has changed the face and shape of higher education as, for one, it has accelerated the addition of remote learning for traditionally residential universities (Srivastava et al., 2020). As remote learning may now be permanently added to the job description of academics, renegotiation of the contract, and more specifically, the psychological contract is needed. Lastly, relational terms should be included in the psychological contract. Collaboration and team efforts were found to be critical to managing the COVID-19 crisis period in higher education (du Plessis et al., 2022). Thus, utilizing collaborative approaches to navigate the post-pandemic higher education landscape will be essential to re-engage academics and their institutions for the future.

### Limitations

The dataset analyzed for this study come from a wider study of women academics in South African universities. As a result, the findings may reflect the unique aspects of higher education in South African and the lockdown conditions instituted by the South African government. While women academics were purposively sampled for this research, the study would have benefitted from the inclusion of male academics to gauge the gendered nature of the experiences. In addition, other demographic categories, such as career stages, disciplines, and geographical sites, may be relevant differentiators that future studies could explore. Although the open-ended research instrument allowed for rich descriptions of individual experiences, an in-depth qualitative study exploring how women academics have attempted to repair their psychological contract might be a promising future research direction.

## Conclusion

Our findings show that the relationships between women academics and their institutions were reshaped during the COVID-19 lockdown period through a series of mostly unmet expectations. Aspects such as low levels of communication; the added workload—seemingly unappreciated by institutions—and resultant pressure of extra tasks; the inadequate provision of resources to deal with online student interaction and to continue to engage with colleagues; and a seemingly poorly prepared leadership response, resulted in a shift in the psychological contract.

While there are some limitations in the study, we believe that the acknowledgment of breaches and violation of the psychological contract opens up opportunities for trust building and renegotiation. As such, recommendations were offered to renegotiate the psychological contract between academics and their institutions in the post-pandemic higher education landscape.

## Data availability statement

The datasets presented in this article are not readily available because, as part of the agreement for access with all universities, we undertook to keep the dataset completely confidential. Requests to access the datasets should be directed to CW, cyrillwalters@sun.ac.za.

## Ethics statement

The studies involving human participants were reviewed and approved by the Stellenbosch University Ethics Committee. The patients/participants provided their written informed consent to participate in this study.

## Author contributions

LR and MP conceptualized the manuscript’s focus, proposed the objectives, prepared the draft manuscript, and wrote all the sections. CW conceptualized the original broader study from which the data were drawn and collected all the data. All authors contributed to the article and approved the submitted version.
